# Association between Cardiorespiratory Fitness and Health-Related Quality of Life among Patients at Risk for Cardiovascular Disease in Uruguay

**DOI:** 10.1371/journal.pone.0123989

**Published:** 2015-04-22

**Authors:** Morgan N. Clennin, Jonathan P. W. Payne, Edgardo G. Rienzi, Carl J. Lavie, Steven N. Blair, Russell R. Pate, Xuemei Sui

**Affiliations:** 1 Department of Exercise Science, Arnold School of Public Health, University of South Carolina, Columbia, South Carolina, United States; 2 Especialista en Medicina del Deporte, Centro Calidad de Vida, Asociación Española, Montevideo, Uruguay; 3 Department of Cardiovascular Diseases, John Ochsner Heart and Vascular Institute, Ochsner Clinical School-the University of Queensland School of Medicine, New Orleans, Louisiana, United States; 4 Department of Preventive Medicine, Pennington Biomedical Research Center, Louisiana State University System, Baton Rouge, Louisiana, United States; 5 Department of Epidemiology and Biostatistics, Arnold School of Public Health, University of South Carolina, Columbia, South Carolina, United States; Universidad Pablo de Olavide, Centro Andaluz de Biología del Desarrollo-CSIC, SPAIN

## Abstract

To date, few studies have examined the relationship between cardiorespiratory fitness (CRF) and health-related quality of life (HRQoL) in populations at high risk for developing cardiovascular disease (CVD).

**Purpose:**

To examine the association between objectively measured CRF and physical and mental components of HRQoL in a Uruguayan cohort at risk for developing CVD.

**Methods:**

Patient data records from 2002–2012 at the Calidad de Vida Center were examined. To assess CRF, participants performed a submaximal exercise test. During the evaluation, participants also completed the SF-36, a HRQoL measure comprised of eight dimensions that are summarized by physical and mental component scores (PCS and MCS, respectively). ANCOVA was used to examine the relationship between HRQoL dimensions and CRF. Logistic regression was then used to compare the odds of having a HRQoL component score above the norm across CRF. All analyses were performed separately for males and females with additional stratified analyses across age and BMI conducted among significant trends.

**Results:**

A total of 2,302 subjects were included in the analysis. Among females, a significant relationship was observed between CRF and vitality, physical functioning, physical role, bodily pain, and general health dimensions. However, for males the only dimension found to be significantly associated with CRF was physical health. After adjusting for potential confounders, a significant linear trend (p<0.001) for PCS scores above the norm across CRF levels was observed for females only.

**Conclusion:**

Among females with one or more risk factors for developing CVD, higher levels of CRF were positively associated with the vitality and physical dimensions of HRQoL, as well as the overall PCS. However, among males the only dimension associated with CRF was physical functioning. Future studies should examine this relationship among populations at risk for developing CVD in more detail and over time.

## Introduction

Cardiovascular disease (CVD) is the leading cause of death worldwide, accounting for more than 17 million deaths in 2008 alone [1]. While CVD is largely preventable through risk factor modification, the prevalence of CVD is expected to increase dramatically to 23.6 million deaths in 2030, with disproportionate increases experienced in developing countries. The anticipated increase in CVD has been attributed to the rise in risk factors associated with the disease, including hypertension, obesity, and physical inactivity [1,2]. Despite the well-established benefits associated with regular physical activity (PA) [3], globally, a majority of adults are not engaging in the recommended amounts of PA [4,5]. Consequently, these low levels of PA have been identified as a contributing factor to numerous adverse chronic health conditions (e.g., coronary heart disease, diabetes, and stroke), all-cause mortality, and an overall decrease in health-related quality of life (HRQoL) [5–8].

HRQoL is defined as an individual’s perception of their overall physical, mental, and social health with respect to their current health and can provide a better understanding of an individual’s overall health status [9]. From a public health perspective, HRQoL measures provide a better understanding of how HRQoL can be influenced by interventions that promote improvements in health enhancing lifestyle behaviors, such as PA. Previous research has employed economic analyses using the associations between HRQoL and varying health states to better inform decisions pertaining to the allocation of available resources [10,11]. Ultimately, understanding these relationships can help guide resource allocation to effective prevention programs and policies that will result in the greatest impact on the population’s health.

To date, many studies have examined the association between HRQoL and PA with results suggesting a positive relationship [12–14]. However, substantially less research has examined the relationship between HRQoL and cardiorespiratory fitness (CRF), a measure the body’s capacity to transport and utilize oxygen [12,15–17]. While limited, previous literature suggests, in general, that a positive association between HRQoL and CRF exists; however, the mechanisms explaining this relationship are still debated. Some researchers have proposed CRF as a possible mediator of the relationship between PA and HRQoL, as CRF is influenced, in part, by habitual PA [16–18]. Other researchers have suggested that despite being moderately correlated, PA and CRF may be partially independent of one another and represent different constructs [19]. Notably, research supporting this relationship has been conducted among older and chronically ill populations [12, 20, 21] as well as younger and generally healthy populations [12, 22, 23]. Currently, however, there is insufficient evidence available regarding the relationship between CRF and HRQoL among individuals at high risk for developing CVD.

To address the above gaps in the literature, the present study aims to explore the relationship between CRF and a broad measure of health status in a clinical population at risk for developing CVD. Our operational definition of “at risk” is individuals with one or more risk factors and/or co-morbidities for developing CVD, including hypertension, dyslipidemia, pre-diabetes or diabetes, overweight/obesity, smoking, and a sedentary lifestyle [24, 25]. To the best of our knowledge, no previous studies have examined the relationship between CRF and HRQoL in individuals at high risk of developing CVD. We hypothesized that higher levels of CRF would be associated with higher self-reported HRQoL among individuals with one or more risk factors for CVD. With the increasing rates of CVD [8], understanding the HRQoL among populations at high risk for developing CVD and its association with CRF should be prioritized. Hence, the objective of this cross-sectional analysis is to examine, by sex, the association between objectively measured CRF level and the physical and mental health components of HRQoL in a Uruguayan population at risk for developing CVD.

## Methods

### Data Source

The data were obtained from the Calidad de Vida Center, a Health Department of the Spanish Association in Uruguay. The center’s primary focus is to work with individuals with one or more risk factors for developing CVD to develop targeted interventions designed to control risk factors through improvements in lifestyle health behaviors. All patients were referred to the center by their primary health care provider after being identified as at increased risk for developing CVD based on their current health status and lifestyle behaviors. Specifically, individuals referred to the center had one or more risk factors for developing CVD, including but not limited to hypertension, dyslipidemia, pre-diabetes or diabetes, smoking, and a sedentary lifestyle. Licensed medical professionals conducted a detailed health assessment during each patient’s initial appointment. All tests were conducted by the clinic’s staff using standard medical practices which were approved by the center’s ethics committee. Patients referred to the Calidad de Vida Center provided written informed consent accepting the risks associated with exercise and the possibility of their information being used for scientific purposes.

### Participants

For these analyses, data records from patients referred to the Calidad de Vida Center by a health care provider from January 1, 2002—December 31, 2012 were examined. Of the patient data records available, only records containing complete data for CRF, HRQoL, and covariates of interests (i.e., age, sex, body mass index (BMI), etc.) were included in the analyses. Additionally, the analyses were restricted to adult patients; hence, patient data records for individuals less than 18 years of age were excluded from the analyses. Of the 2,772 unique records identified, 2,302 (83.0%) contained required data and met the inclusion criteria.

### Cardiorespiratory Fitness

Patients performed a graded exercise test on a treadmill using the modified Bruce protocol under the supervision of a licensed cardiologist. The modified Bruce protocol starts at 1.7 mph/2.74 kph and 0 percent grade and progresses to a new stage every three minutes. The submaximal exercise test was conducted and terminated in accordance with American College of Sports Medicine safety guidelines [24, 26]. Standard tables were used to estimate metabolic equivalent (MET) levels based on participants’ grade and speed when the test was terminated [26]. Participants were categorized into quartiles based on age- (18–29; 30–39; 40–49; 50–59; 60–69; 70+) and sex-specific distributions of the estimated maximal MET levels reported from the submaximal CRF exercise test.

### Health-Related Quality of Life

HRQoL is an individual’s perception of their overall life satisfaction and can typically be summarized by the functional status among several health domains including physical, cognitive, emotional, and social health [9]. To assess HRQoL, the Medical Outcomes Study SF-36 Quality of Life Scale, a generic health status instrument, was self-administered to each participant during their initial visit to the clinic [9]. The 36-item instrument is reliable and valid and used widely across both ill and healthy populations [27–29]. In total, the instrument yields eight subscales of health (physical function, physical role, bodily pain, general health, vitality, social functioning, emotional role, and mental health) and two component summary scores (physical and mental; PCS and MCS, respectively). The questionnaire is scored via an established protocol employing norm based scoring algorithms. Using the established scoring protocol, the eight domain scores were calculated and then summarized into PCS and MCS summary scales ranging from 0 to 100 [28, 30], with higher scores representing better wellbeing and higher functioning. The general population has a mean of 50 and a standard deviation of 10 for each component summary score. Hence, a score below 50 is considered to be below the norm when compared to the general population [31].

### Statistical Analysis

Analyses were conducted separately for males and females. Using the estimated maximal MET levels from the graded exercise test, participants were categorized into quartiles. Descriptive statistics were examined across the entire population by sex and fitness quartiles. To examine the potential dose-response relationship between HRQoL dimensions and CRF, separate ANCOVAs adjusting for potential covariates were conducted by gender for each dimension. In addition, logistic regression was used to compare the odds of having a HRQoL PCS or MCS above the norm across fitness groups, with the least fit quartile serving as the reference group. Fully-adjusted models included the following covariates: age, BMI, hypertension (yes or no), current smoking status (yes or no), and physically inactive (yes or no). Patients self-reporting less than 30 minutes of daily PA on three days per week (150 minutes/week) were classified as physically inactive. We assessed linear and non-linear trends in the association of CRF with HRQoL dimensions and component scores in both males and females. Among significant trends in PCS and MCS, we also performed stratified analyses across age (<65 and ≥65 years) and BMI (<30 and ≥30) to assess whether these two factors modified the association between fitness and each outcome. All statistical analyses were conducted using SAS 9.3 and probability values less than 0.05 were considered statistically significant.

## Results

Table 1 depicts the demographic characteristics of male (n = 1,000) and female (n = 1,302) subjects according to CRF levels. On average, subjects were middle-aged (~55 years), overweight/obese, and predominately inactive with 82.9 percent of males and 86.8 percent of females failing to meet PA guidelines. The proportion of women with a mean PCS and MCS above 50 (66.6% and 69.6%, respectively) was lower compared to male subjects (85.6% and 83.9%, respectively). Across CRF quartiles, estimated maximal METS, PCS and MCS increased while BMI, and the prevalence of hypertension and physical inactivity decreased. In addition, HRQoL dimension scores generally increased across CRF quartiles, S1 Table. Among females and males, weak correlations existed between raw PCS vs. METs (r = 0.24 and r = 0.23, respectively) and MCS vs. METs (r = 0.03 and r = 0.01, respectively).

**Table 1:** Participant demographic characteristics by gender and cardiorespiratory fitness level.

Sex	Characteristics^b^	Cardiorespiratory Fitness Levels, quartiles^a^				
		All	Q1 (Ref)	Q2	Q3	Q4
**Females**	(n)	1302	332	319	332	319
	Age (years)	55.0 (12.7)	55.4 (12.4)	55.2 (12.3)	55.2 (12.5)	54.4 (13.7)
	BMI (kg*m^2^)	31.4 (6.4)	35.6 (6.7)	30.7 (5.5)	29.5 (6.0)	29.6 (5.4)
	Current Smoker (%)	14.7	13.3	15.7	18.4	11.3
	Hypertensive (%)	49.1	58.4	44.8	45.2	47.6
	Physically Inactive (%)	86.8	94.0	89.0	85.2	78.7
	Ergometry METS	5.9 (1.9)	3.9 (0.6)	5.0 (0.5)	6.2 (0.7)	8.4 (1.4)
	PCS	64.4 (17.9)	58.7 (19.4)	64.9 (17.3)	65.3 (17.8)	68.7 (15.9)
	MCS	64.8 (20.0)	63.2 (19.3)	66.2 (19.4)	63.2 (21.4)	66.6 (19.7)
**Males**	(n)	1000	248	250	248	254
	Age (years)	55.5 (12.7)	55.3 (12.9)	55.8 (12.7)	56.2 (12.9)	54.8 (12.5)
	BMI (kg*m^2^)	31.6 (5.6)	35.1 (6.3)	31.3 (5.0)	30.5 (4.9)	29.6 (4.1)
	Current Smoker (%)	18.2	23.0	18.8	14.1	16.9
	Hypertensive (%)	61.3	68.6	58.8	58.1	59.8
	Physically Inactive (%)	82.9	91.5	81.6	81.5	77.2
	Ergometry METS	7.2 (2.4)	4.5 (0.8)	6.1 (0.7)	7.7 (1.1)	10.4 (1.4)
	PCS	73.3 (14.8)	68.7 (16.0)	72.7 (14.6)	74.5 (14.2)	77.2 (13.2)
	MCS	72.5 (17.8)	73.1 (17.1)	72.2 (17.2)	71.1 (19.4)	73.8 (17.4)

BMI, body mass index, METs, metabolic equivalents, PCS, physical component score, MCS, mental component score, Q, quartile.

^a^Cardiorespiratory fitness level quartiles established using age- and sex-specific MET values.

^b^Data listed as Mean (SD) unless otherwise specified.

The adjusted HRQoL physical and mental component scores as well as individual HRQoL dimension scores are reported in Table 2 by gender and CRF quartile. After adjusting for potential covariates (age, BMI, hypertension, current smoking status, and physical inactivity), significant linear trends for overall PCS were observed for both females (p-value for linear trend <0.0001) and males (p-value for linear trend <0.0001). Among females, a significant linear relationship was observed across fitness levels for vitality (p<0.003) as well as each of the four physical dimensions, physical health (p<0.001), physical role (p<0.09), bodily pain (p<0.001), and general health (p<0.001). For males, the physical health dimension (p <0.001) was the only dimension to show a significant linear trend across fitness quartiles. Non-linear trends were examined; results revealed a significant non-linear trend across CRF levels for MCS (p = 0.031) and social function (0.004) among males.

**Table 2:** Adjusted health-related quality of life (HRQoL) dimension scores by gender and cardiorespiratory fitness level.

Sex	HRQoL		Cardiorespiratory Fitness Levels, quartiles^a^				p-value for linear trend
			Q1	Q2	Q3	Q4	
**Females**	**(n)**		332	319	332	319	
	**PCS** ^b^		60.8 (1.2)	65.6 (1.2)	65.6 (1.1)	68.9 (1.2)	<0.001
		Physical Health	63.8 (1.3)	68.2 (1.3)	69.5 (1.2)	73.6 (1.3)	<0.001
		Physical Role	67.3 (2.6)	72.7 (2.5)	68.8 (2.4)	73.4 (2.5)	0.092
		Bodily Pain	52.5 (1.7)	57.8 (1.7)	60.7 (1.6)	60.4 (1.7)	<0.001
		General Health	52.9 (1.2)	57.9 (1.2)	57.3 (1.2)	59.3 (1.2)	<0.001
	**MCS**		62.0 (1.4)	65.3 (1.4)	62.4 (1.3)	65.4 (1.3)	0.170
		Vitality	47.1 (1.4)	53.1 (1.4)	52.3 (1.4)	52.8 (1.4)	0.003
		Social Function	73.6 (1.7)	77.3 (1.7)	72.6 (1.6)	76.8 (1.7)	0.464
		Emotional Role	74.1 (2.6)	76.5 (2.5)	70.3 (2.4)	75.4 (2.5)	0.821
		Emotion Health	60.8 (1.4)	63.2 (1.3)	61.7 (1.3)	64.5 (1.3)	0.069
**Males**	**(n)**		248	250	248	254	
	**PCS**		71.0 (1.1)	73.2 (1.0)	74.5 (1.1)	76.6 (1.1)	<0.001
		Physical Health	72.8 (1.2)	76.8 (1.1)	80.3 (1.2)	82.6 (1.2)	<0.001
		Physical Role	80.4 (2.3)	82.1 (2.3)	78.7 (2.3)	83.2 (2.3)	0.591
		Bodily Pain	73.7 (1.7)	72.2 (1.6)	73.7 (1.7)	73.9 (1.6)	0.758
		General Health	59.0 (1.3)	59.5 (1.3)	59.9 (1.3)	60.4 (1.3)	0.387
	**MCS**		73.7 (1.4)	71.4 (1.3)	69.7 (1.4)	72.4 (1.4)	0.298
		Vitality	60.3 (1.5)	59.4 (1.4)	58.4 (1.5)	61.6 (1.4)	0.603
		Social Function	84.0 (1.6)	81.2 (1.5)	81.7 (1.5)	86.5 (1.5)	0.201
		Emotional Role	85.7 (2.5)	85.5 (2.4)	79.1 (2.5)	81.4 (2.5)	0.086
		Emotion Health	71.4 (1.4)	69.5 (1.3)	69.0 (1.4)	70.4 (1.4)	0.458

PCS, physical component score, MCS, mental component score, Q, quartile.

^a^Cardiorespiratory fitness level quartiles (Q1-Q4) established using age- and sex-specific MET values.

^b^Data listed as Mean (SD) adjusted for age, body mass index, hypertension, current smoking status, and physical inactivity unless otherwise specified.

Table 3 shows the odds ratios (ORs) and 95% confidence intervals (CIs) for above the norm (score of 50) PCS and MCS across CRF levels. Individuals in the lowest CRF group served as the referent group. Adjusting for age, significant positive trends across CRF levels were observed among females and males for the ORs of PCS above the median (p<0.0001 and p = 0.007; respectively), while no significant trends were observed across MCS. After adjusting for potential confounding variables, the significant trends observed among PCS above the median across CRF groups persisted in females only (p<0.0001). Based on this significant trend among females, results indicate that lower levels of CRF were associated with lower PCS after adjusting for age, BMI, hypertension, current smoking status, and physical inactivity. For males, no significant trends across CRF levels for the odds of PCS and MCS above the median were observed after adjusting for potential confounders. Hence, all subsequent analyses were performed among females only.

**Table 3:** Odds ratios (ORs) above the norm for PCS and MCS according to cardiorespiratory fitness level.

Sex	HRQoL		Cardiorespiratory Fitness Levels, quartiles^a^				p-value for linear trend
			Q1 (Ref)	Q2	Q3	Q4	
**Females**	(n)		332	319	332	319	
	**PCS** ^b^	PCS ≥50	66.6%	78.4%	76.2%	85.9%	—
		Age-Adjusted OR	1.00	1.82 (1.28–2.59)	1.61 (1.15–2.27)	3.04 (2.06–4.50)	<0.001
		Multivariate OR^c^	1.00	1.66 (1.14–2.40)	1.43 (0.99–2.07)	2.68 (1.77–4.05)	<0.001
	**MCS**	MCS ≥50	69.6%	76.8%	70.1%	75.9%	—
		Age-Adjusted OR	1.00	1.45 (1.02–2.06)	1.03 (0.74–1.43)	1.38 (0.97–1.95)	0.256
		Multivariate OR^c^	1.00	1.48 (1.03–2.14)	1.06 (0.74–1.51)	1.40 (0.96–2.03)	0.288
**Males**	(n)		248	250	248	254	
	**PCS**	PCS ≥50	85.6%	93.6%	91.9%	93.7%	—
		Age-Adjusted OR	1.00	2.47 (1.32–4.60)	1.94 (1.08–3.48)	2.42 (1.30–4.52)	0.007
		Multivariate OR^c^	1.00	1.60 (0.83–3.09)	1.19 (0.62–2.26)	1.32 (0.65–2.66)	0.569
	**MCS**	MCS ≥50	83.9%	82.4%	80.6%	85.0%	—
		Age-Adjusted OR	1.00	0.90 (0.57–1.45)	0.81 (0.51–1.28)	1.09 (0.67–1.77)	0.870
		Multivariate OR^c^	1.00	0.84 (0.51–1.37)	0.72 (0.44–1.17)	0.95 (0.57–1.60)	0.737

ORs, odds ratios, PCS, physical component score, MCS, mental component score.

^a^Cardiorespiratory fitness level quartiles established using age- and sex- specific MET values.

^b^Data presented as odds ratio (OR) and 95% confidence interval unless otherwise specified.

^c^adjusted for age, body mass index, hypertension, current smoking status, and physical inactivity.

**Fig 1:**
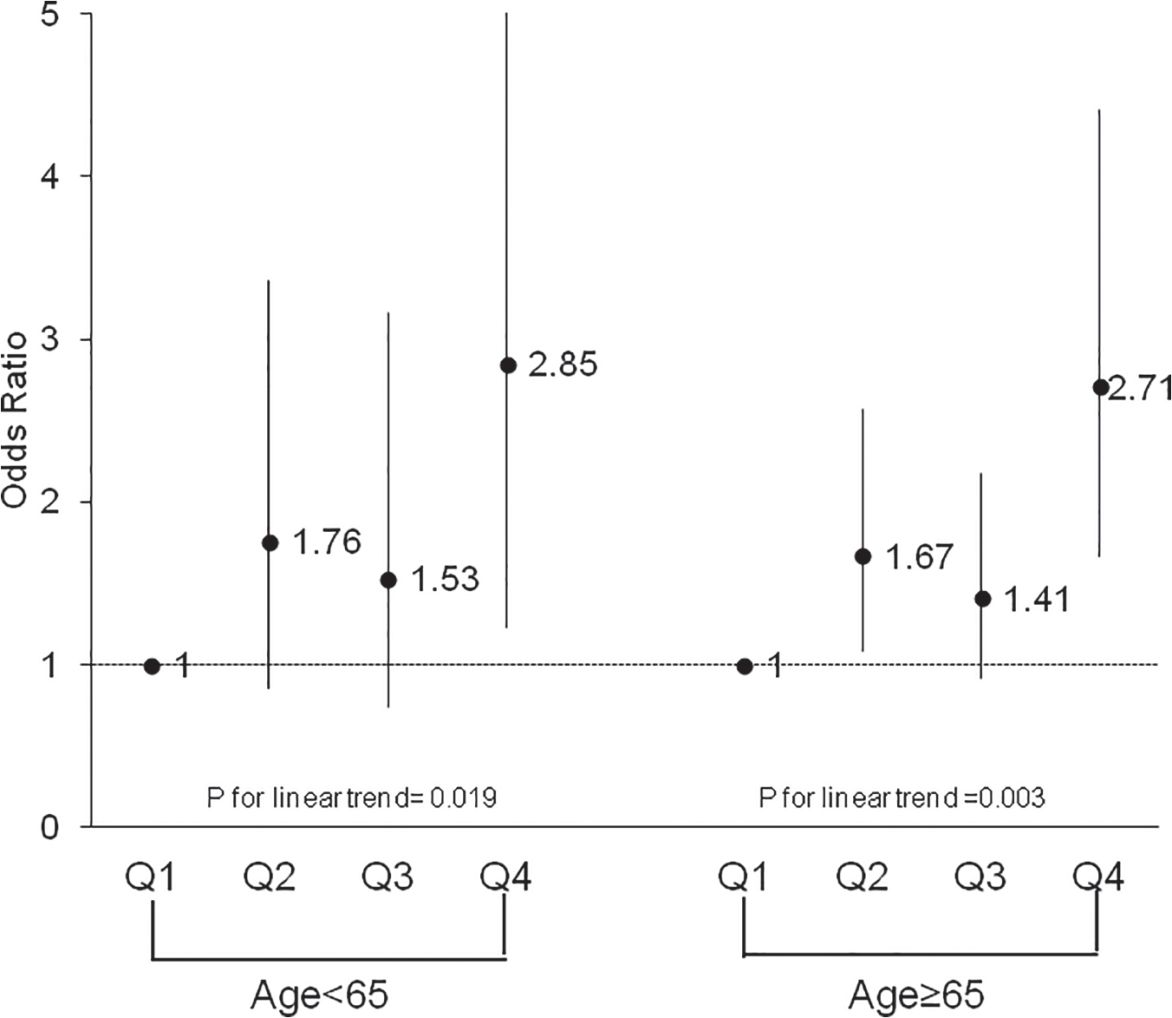
Odds ratios above the norm for health-related quality of life physical component score according to cardiorespiratory fitness quartile and age group among females. Logistic regression adjusting for age, body mass index, smoking status, hypertension, and physical inactivity was used to obtain odds ratios and the p-value for the trend analysis, Q, quartile.

**Fig 2:**
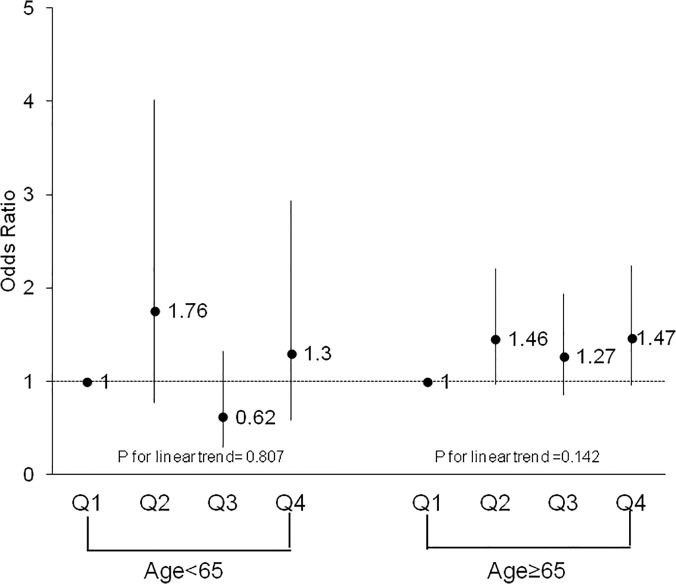
Odds ratios above the norm for health-related quality of life mental component score according to cardiorespiratory fitness quartiles and age group among females. Logistic regression adjusting for age, body mass index, smoking status, hypertension, and physical inactivity was used to obtain odds ratios and the p-value for the trend analysis, Q, quartile.

**Fig 3:**
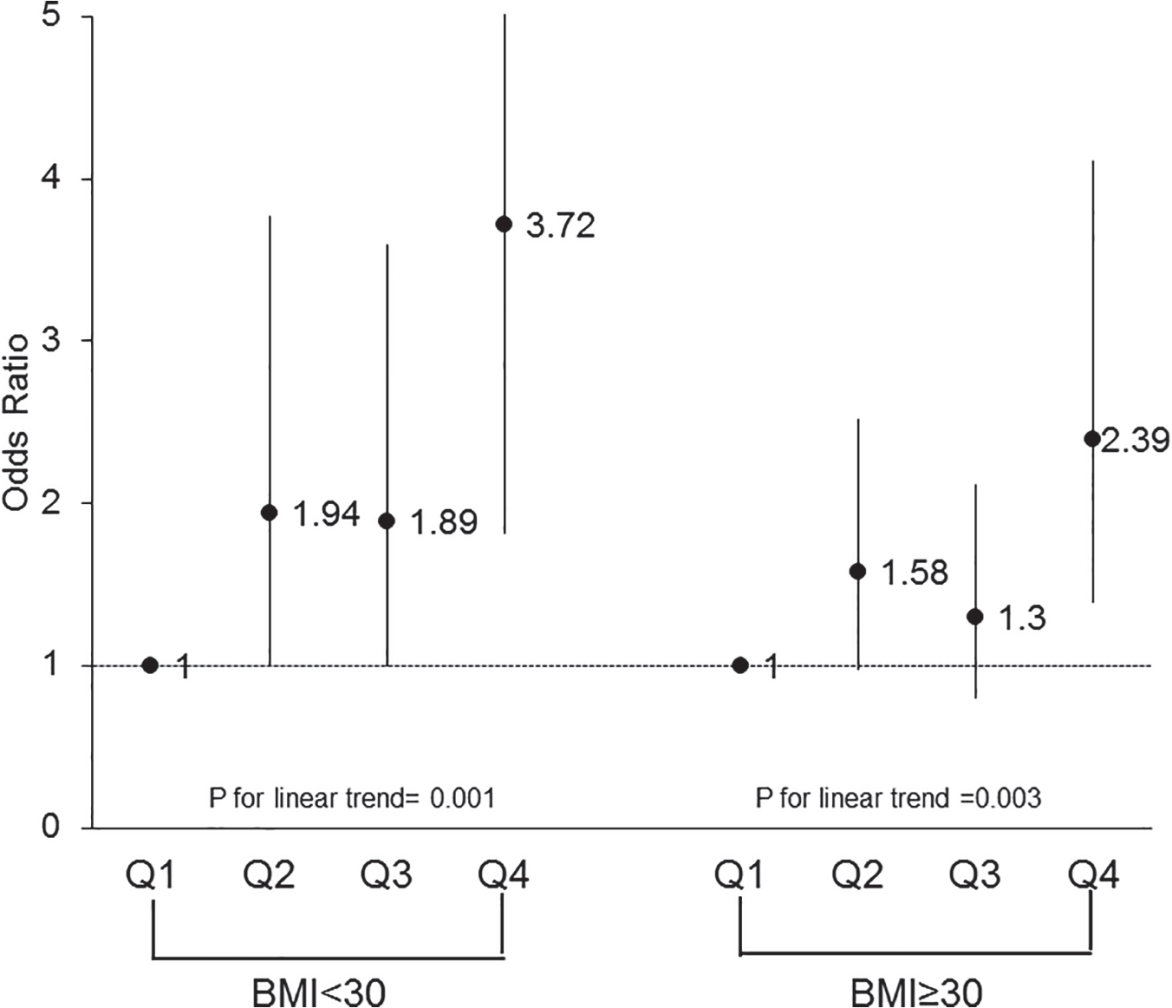
dds ratios above the norm for health-related quality of life physical component score according to cardiorespiratory fitness levels and body mass index among females. Logistic regression adjusting for age, body mass index, smoking status, hypertension, and physical inactivity was used to obtain odds ratios and the p-value for the trend analysis, Q, quartile.

**Fig 4:**
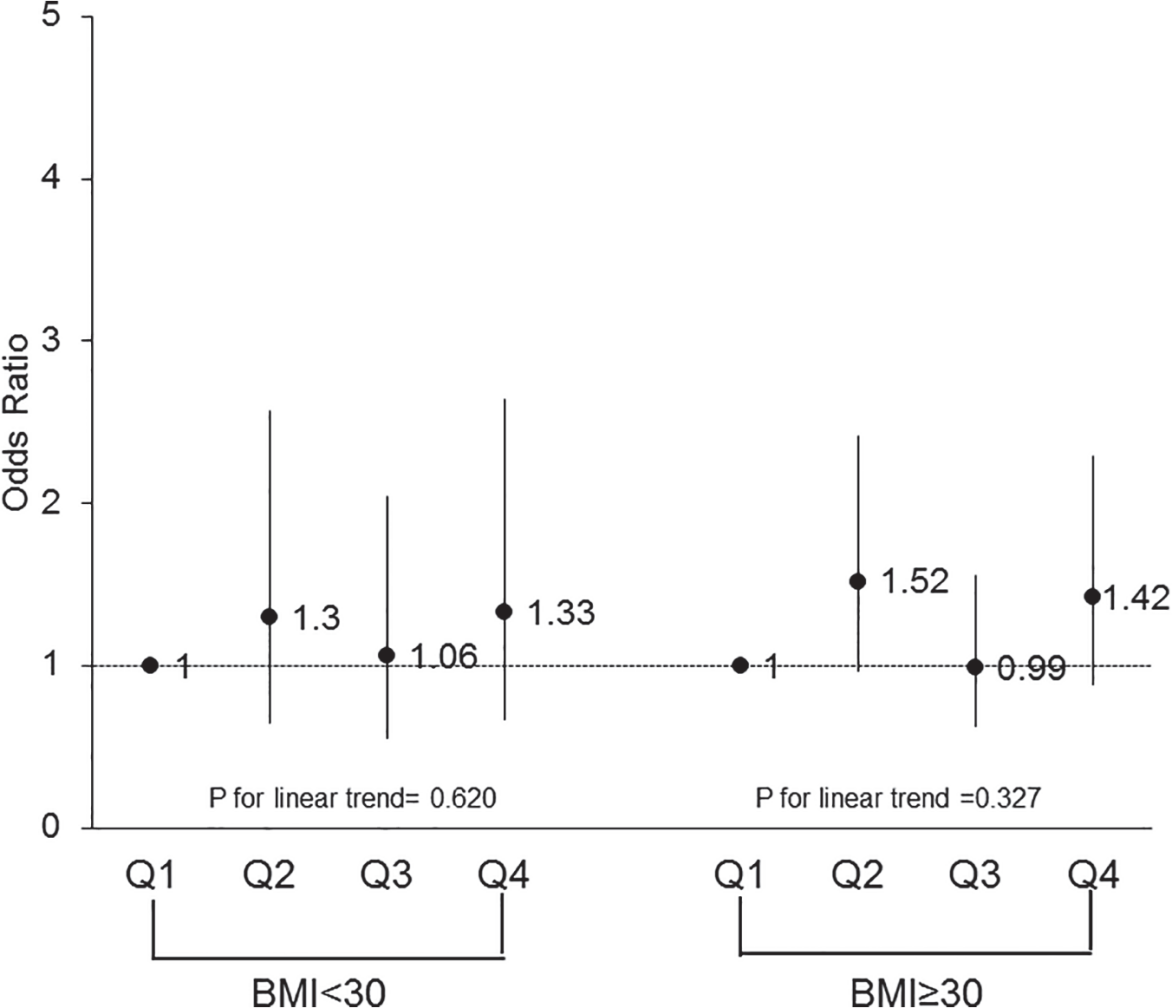
Odds ratios above the norm for health-related quality of life mental component score according to cardiorespiratory fitness levels and body mass index among females. Logistic regression adjusting for age, body mass index, smoking status, hypertension, and physical inactivity was used to obtain odds ratios and the p-value for the trend analysis, Q, quartile.

To further examine the association between CRF and HRQoL component scores, females were split into groups based on age (<65 years old vs. ≥65 years old) and obesity status (obese: BMI ≥30 vs. non-obese: BMI <30). Both age groups were similar in demographic composition. Comparing older women (≥65 years old) to younger women (<65 years old), the significant trend across CRF for multivariate-adjusted ORs for PCS above the norm persisted across both age groups (p = 0.0003 and p = 0.02, respectively). The trend for MCS scores above the median remained non-significant across both older and younger women (p = 0.81 vs. 0.14, respectively). Figs 1 and 2 depict these trends across CRF groups for both age groups. Stratifying females by obesity status, a significant trend across CRF quartiles for PCS was observed for both obese (p = 0.003) and non-obese (p = 0.001) groups after adjusting for potential covariates. Similar to previous results, the trend for MCS above the norm remained non-significant when stratified by obesity status (obese: p = 0.33; non-obese: p = 0.62). Figs 3 and 4 depict the trends in the ORs across CRF among obese and non-obese women.

## Discussion

This study examined the HRQoL among an Uruguayan population at risk for developing CVD and its association with CRF. Our results suggest that there is a positive relationship between some dimensions of HRQoL and CRF; however, these relationships were found to differ by sex. For females, a significant trend across fitness quartiles was observed for five of the eight HRQoL dimensions, including vitality and the four physical dimensions. Fewer HRQoL dimensions were associated with CRF among males with only the physical health dimension revealing a significant positive relationship with CRF. However, non-linear trends across CRF levels for the MCS and social functioning dimension were observed for males indicated a different relationship may exist. Among both males and females, a significant dose-response relationship across fitness was observed for the PCS; however, no relationships were found between CRF and the MCS of HRQoL after controlling for potential covariates. One possible explanation for the observed difference in the relationship between HRQoL and CRF across gender could be a difference in the perception of HRQoL. More specifically, it is possible that females and males respond differently to the HRQoL questionnaire due to a discrepancy in how quality of life is perceived by females compared to males.

The present study supports several previous studies that have reported a positive relationship between CRF and the HRQoL dimensions physical functioning, physical role, bodily pain, and vitality [32–35]. Conversely, another recent study examining the relationship between CRF and HRQoL among a small sample of healthy, normal weight adults found no significant associations between CRF and any dimensions of HRQoL after adjusting for age and BMI [36]. However, this discrepancy is likely attributable to the small sample size, different population studied, and/or a difference in statistical methodology and covariates. Similar to our findings, some literature has identified a dose-response relationship between fitness and several dimensions of HRQoL. Specifically, Erikson et al (2013) reported a strong dose-response relationship between CRF and self-rated health for both males and females [37], while Perales et al (2014) observed a positive dose-response relationship between moderate-to-vigorous PA and all dimensions of the SF-36, with the strongest relationships observed between activity level and the physical health and vitality HRQoL dimensions [38]. While the present study did observe similar dose-response relationships between several dimension of HRQoL and CRF (females: vitality, physical health, physical role, bodily pain, general health; males: physical health) [32,33,35,39], our results indicate a stronger relationship among females compared to males, which has not been reported in previous studies.

Concerning the odds of having a PCS and MCS above the population median, our results suggest that there is a positive relationship between CRF and the PCS part of HRQoL among females; however, no association was observed between CRF level and the MCS for this population. Subsequent analyses conducted in the female population to examine the associations between HRQoL component scores and CRF revealed similar trends when stratified by age and BMI. Among males, the odds of having a PCS or MCS above the norm did not differ across fitness quartiles. In comparison, some studies support a positive associations between CRF for both PCS and MCS [20,31,33,40], while others have reported null findings [36]. For instance, Rejeski and colleagues (2006) reported a relationship between lower PCS and lower CRF levels among middle-aged overweight/obese adults with Type 2 Diabetes, which aligns with the results of the current study [20]. However, this relationship was observed among women only in the present study.

In general, our results support previous literature which has reported higher levels of CRF to be associated with higher levels of HRQoL in younger males [31,40], the general population [12,33,36,38], elderly [32,41–42], postmenopausal women [43], and in chronically ill populations [20,21,44]. However, the magnitude of this relationship varies across studies, dimensions of HRQoL, and type of measure used (objective vs. subjective). These inconsistencies between our results and other studies noted above may be due to variations in fitness measures used (PA vs. CRF) as well as the difference in study populations as this is the first study to specifically examine the relationship between HRQoL and CRF in a population at risk for developing CVD.

To the best of our knowledge, this is the first study to evaluate the association between CRF level and HRQoL in a clinical population at risk of developing CVD (i.e., one or more risk factors and/or co-morbidities for CVD). Despite the growth in evidence supporting this relationship, many researchers note that the relationship between HRQoL and physical fitness has not been examined sufficiently to draw definitive conclusions [40,45]. In particular, the physiological mechanisms explaining the relationship between CRF and HRQoL are not fully understood. Previously literature has proposed several physiological mechanisms influencing the relationship between PA and HRQoL [10,46]. Specifically, habitual PA has been shown to increase CRF [17,18] as well as improve some biological risk factors related to physical and mental health including a reduction in dyslipidemia, glucose intolerance, vascular dysfunction, inflammation, as well as depression and anxiety symptoms [39,47]. While PA and CRF are moderately correlated [19], the mechanisms implicated in the relationship between PA and HRQoL may differ from those implicated in the CRF and HRQoL relationship, hence, more research is needed to explore these relationships.

Several strengths and limitations should be addressed for the present study. The large clinical population examined provided adequate power to examine the relationship between components of HRQoL and CRF. A second strength included the use of a HRQoL instrument which employs norm based scoring allowing for comparisons between other studies evaluating HRQoL, regardless of the population studied or the version of the SF-36 used. Finally, to the author’s knowledge, this is the first study to examine the relationship between objectively measured CRF levels and HRQoL among individuals at risk of developing CVD; hence the authors believe the present study addresses an important gap in the literature by examining this population.

One of the primary limitations of this analysis was the use of cross-sectional data to assess the relationship between CRF level and HRQoL. The use of this study design limits our ability to establish temporality, or more specifically, our ability to determine if a direct cause and effect relationship exists between CRF and HRQoL. Further, the generalizability of this study may be limited due to the sample examined (i.e., high risk CVD population with access to health care in Uruguay). Due to the limited research in populations at risk for developing chronic conditions such as CVD, more studies should be conducted to assess this relationship. Additionally, the authors were not able to examine the influence of additional demographic characteristics as this information was not included in the patient data records (i.e., education, martial status, socioeconomic position, etc.), and therefore could not be controlled for in the analyses. We recommend that future studies include such variables into their analyses if possible. The measures used for the present study also presented limitations. Specifically, the use of a self-reported HRQoL instrument could introduce reporting biases resulting in an over- or underestimation of the relationship between HRQoL and CRF. However, as HRQoL is an individual’s perception of their current health status, an objective measure is not available. A final limitation to note for the current study is the use of a submaximal exercise test to assess CRF and estimate maximal MET levels. However, for the given population, this test was appropriate and has been identified as a valid method for assessing CRF by the American Heart Association [26].

### Conclusions

In conclusion, the results of the present study suggest that among females, lower levels of CRF are associated with lower levels of self-reported physical and vitality dimensions but not mental health when using a validated HRQoL measurement tool. Notably, this relationship did not hold true for males across most HRQoL dimension and the PCS or MCS components. Stratification of the female population by age and BMI revealed similar trends in the relationship between PCS and MCS components of HRQoL and CRF. Future studies should employ prospective study designs to assess the cause and effect relationship between CRF and HRQoL among diverse populations. Additional randomized controlled trials are needed to examine the dose-response relationship between exercise training, CRF, and HRQoL measures in greater detail as well as the underlying physiological and psychological mechanisms influencing this relationship. Ultimately, a clear understanding of the relationship between CRF and HRQoL can lead to the prioritization of effective public health programs. Specifically, if future studies can establish a positive relationship between fitness and HRQoL, economic analyses designed to inform policy development and resource allocation can emphasize the importance of prescribing exercise to patients at risk for developing CVD and the benefits of an exercise referral scheme.

## Supporting Information

S1 File Supplementary STROBE Checklist. (DOC)

S1 Table Health-related quality of life dimension and component scores by gender and cardiorespiratory fitness level. Health-related quality of life dimension scores, and physical (PCS) and mental (MCS) component scores (Mean, SD) by gender and cardiorespiratory fitness quartiles (Q1-Q4).
(DOCX)
