# Supplementary material for: Association between Cardiorespiratory Fitness and Health-Related Quality of Life among Patients at Risk for Cardiovascular Disease in Uruguay
Source: PLoS One. 2015 Apr 22;10(4):e0123989. doi: 10.1371/journal.pone.0123989 (PMC4406735; doi:10.1371/journal.pone.0123989)
Supplement: S1 Table — Health-related quality of life dimension scores, and physical (PCS) and mental (MCS) component scores (Mean, SD) by gender and cardiorespiratory fitness quartiles (Q1-Q4). (DOCX) [file pone.0123989.s002.docx]

**S1 Table. Health-related quality of life dimension and component scores by gender and cardiorespiratory fitness level.**

|  | Cardiorespiratory Fitness Levels, quartiles^a^ | | | | |
| --- | --- | --- | --- | --- | --- |
| HRQoL^b^ | All | Q1 (Ref) | Q2 | Q3 | Q4 |
| **FEMALES (n)** | 1302 | 332 | 319 | 332 | 319 |
| PCS | 64.4 (17.9) | 58.7 (19.4) | 64.9 (17.3) | 65.3 (17.8) | 68.7 (15.9) |
| Physical Health | 67.9 (20.3) | 60.2 (22.0) | 67.9 (20.4) | 69.8 (19.0) | 74.1 (17.2) |
| Physical Role | 66.8 (37.4) | 64.3 (38.2) | 68.4 (37.2) | 64.8 (38.4) | 69.7 (35.7) |
| Body Pain | 58.1 (25.7) | 53.4 (24.9) | 57.5 (27.0) | 60.6 (24.3) | 60.8 (26.0) |
| General Health | 57.8 (18.2) | 53.2 (18.7) | 59.3 (17.6) | 58.4 (18.5) | 60.3 (17.2) |
| MCS | 64.8 (20.0) | 63.2 (19.3) | 66.2 (19.4) | 63.2 (21.4) | 66.6 (19.7) |
| Vitality | 52.5 (21.2) | 48.3 (21.4) | 54.1 (20.4) | 53.5 (22.2) | 54.3 (20.2) |
| Social Function | 75.5 (25.0) | 74.2 (24.6) | 77.8 (24.1) | 73.0 (26.7) | 77.5 (24.3) |
| Emotional Function | 74.2 (37.1) | 74.5 (36.8) | 76.4 (36.4) | 70.1 (38.3) | 75.7 (36.8) |
| Emotion Health | 64.5 (20.0) | 63.1 (19.8) | 65.1 (19.7) | 63.3 (20.6) | 66.5 (19.8) |
| **MALES (n)** | 1000 | 248 | 250 | 248 | 254 |
| PCS | 73.3 (14.8) | 68.7 (16.0) | 72.7 (14.6) | 74.5 (14.2) | 77.2 (13.2) |
| Physical Health | 77.5 (17.3) | 69.5 (19.4) | 76.1 (18.2) | 80.5 (14.6) | 83.7 (12.9) |
| Physical Role | 80.3 (31.2) | 78.7 (33.0) | 81.2 (30.2) | 78.1 (31.1) | 82.9 (30.3) |
| Body Pain | 72.1 (22.1) | 71.5 (23.9) | 71.0 (21.0) | 72.6 (21.0) | 73.4 (22.6) |
| General Health | 59.8 (17.3) | 57.8 (17.9) | 59.8 (16.9) | 60.6 (17.2) | 61.1 (17.0) |
| MCS | 72.5 (17.8) | 73.1 (17.1) | 72.2 (17.2) | 71.1 (19.4) | 73.8 (17.4) |
| Vitality | 61.9 (19.3) | 60.5 (19.4) | 61.5 (19.0) | 61.1 (19.9) | 64.4 (19.1) |
| Social Function | 83.2 (20.6) | 82.4 (20.9) | 81.0 (22.2) | 82.1 (21.0) | 87.3 (17.7) |
| Emotional Function | 82.4 (32.6) | 85.0 (31.4) | 83.4 (30.3) | 79.5 (35.2) | 81.9 (33.2) |
| Emotion Health | 70.5 (18.4) | 70.6 (18.7) | 70.0 (18.4) | 70.2 (18.7) | 71.2 (18.0) |

PCS, physical component score, MCS, mental component score, Q, quartile

^a^Cardiorespiratory fitness level quartiles (Q1-Q4) established using age- and sex-specific MET values.

^b^Data listed as Mean (SD) unless otherwise specified
